# AlphaFold 2 and NMR Spectroscopy: Partners to Understand Protein Structure, Dynamics and Function

**DOI:** 10.3389/fmolb.2022.906437

**Published:** 2022-05-17

**Authors:** Douglas V. Laurents

**Affiliations:** Instituto de Química Física Rocasolano, Consejo Superior de Investigaciones Científicas (IQFR/CSIC), Madrid, Spain

**Keywords:** AlphaFold, NMR spectroscopy, Intrisically disordered proteins, Rare conformations, posttranslational modifications

## Abstract

The artificial intelligence program AlphaFold 2 is revolutionizing the field of protein structure determination as it accurately predicts the 3D structure of two thirds of the human proteome. Its predictions can be used directly as structural models or indirectly as aids for experimental structure determination using X-ray crystallography, CryoEM or NMR spectroscopy. Nevertheless, AlphaFold 2 can neither afford insight into how proteins fold, nor can it determine protein stability or dynamics. Rare folds or minor alternative conformations are also not predicted by AlphaFold 2 and the program does not forecast the impact of post translational modifications, mutations or ligand binding. The remaining third of human proteome which is poorly predicted largely corresponds to intrinsically disordered regions of proteins. Key to regulation and signaling networks, these disordered regions often form biomolecular condensates or amyloids. Fortunately, the limitations of AlphaFold 2 are largely complemented by NMR spectroscopy. This experimental approach provides information on protein folding and dynamics as well as biomolecular condensates and amyloids and their modulation by experimental conditions, small molecules, post translational modifications, mutations, flanking sequence, interactions with other proteins, RNA and virus. Together, NMR spectroscopy and AlphaFold 2 can collaborate to advance our comprehension of proteins.

## Background

In 1961, Anfinsen demonstrated that what determines the three dimensional structure of a protein is encoded in its amino acid sequence (Anfinsen et al., 1961). This raised interest in “decoding” since predicting the structure from sequence would be much simpler than undertaking the laborious effect to solve the 3D structure by X-ray crystallography, which in those days was just beginning to reveal the first protein structures (PERUTZ et al., 1960). Interest in the protein folding problem increased when rapid sequencing methods were introduced (Sanger et al., 1977).

One key insight into how proteins fold was provided by Levinthal, who realized that if protein folding were to occur by a random sampling of conformers, then even the folding of a small protein would require more time than the age of the Universe (Levinthal, 1968). Nevertheless, proteins fold quickly, often within seconds. Scientists quickly deduced that Levinthal’s paradox meant that protein folding must involve intermediates which greatly reduce the conformational space that must be searched. Characterizing these intermediates’ structures was seen as a way to solve the problem. Whereas this presented technical challenges as protein folding is highly cooperative and folding intermediates are generally heterogeneous and sparsely populated, one elegant approach involved using proline isomerization to slow the conversion of intermediate species into fully folded protein and H/D labeling coupled with NMR spectroscopy afforded the identification of which elements of secondary structure fold first (Udgaonkar and Baldwin, 1988). A complementary ingenious method based on site-directed mutagenesis and kinetics revealed the order of side chain structurization during folding (Matouschek et al., 1989). Although these investigations provided insight into how proteins fold, no general solution of the protein folding problem was achieved.

With the development of accurate energy functions for protein folding, it became possible to directly simulate the folding process of very small proteins using molecular dynamics methods and a special purpose, massively parallel computer chip (Lindorff-Larsen et al., 2011). This success confirmed the importance of nascent structural elements in funneling the folding process, nevertheless, it is too slow for larger proteins or proteome-level applications.

In contrast to these physicochemical based methods, other scientists sought to use a more biological approach based on garnering structural inferences from evolutionarily related protein sequences (Bashford et al., 1987). This led to the successful modeling using sequences homologous to a known structure (Fiser, 2010). However, the quality of the structural model varies and depends on how similar a sequence is to that of the known structure.

Further advances have come from the field of protein design, which has produced new folds with novel enzymatic functions (Siegel et al., 2015). The results of protein design also revealed new insights into how proteins fold (Baker, 2019), in particular: 1) the speed of folding increases when contacts are closer together along the sequence, 2) the fold is dictated by thermodynamics, not kinetics, 3) designed proteins can be much more stable than natural ones, and 4) the diversity of natural protein folds is small compared to what is possible. Despite this impressive progress, the problem of predicting protein structure from sequence remained unsolved.

### AlphaFold 2 Successfully Predicts Protein Tertiary Structure From Sequence

**FIGURE 1:**
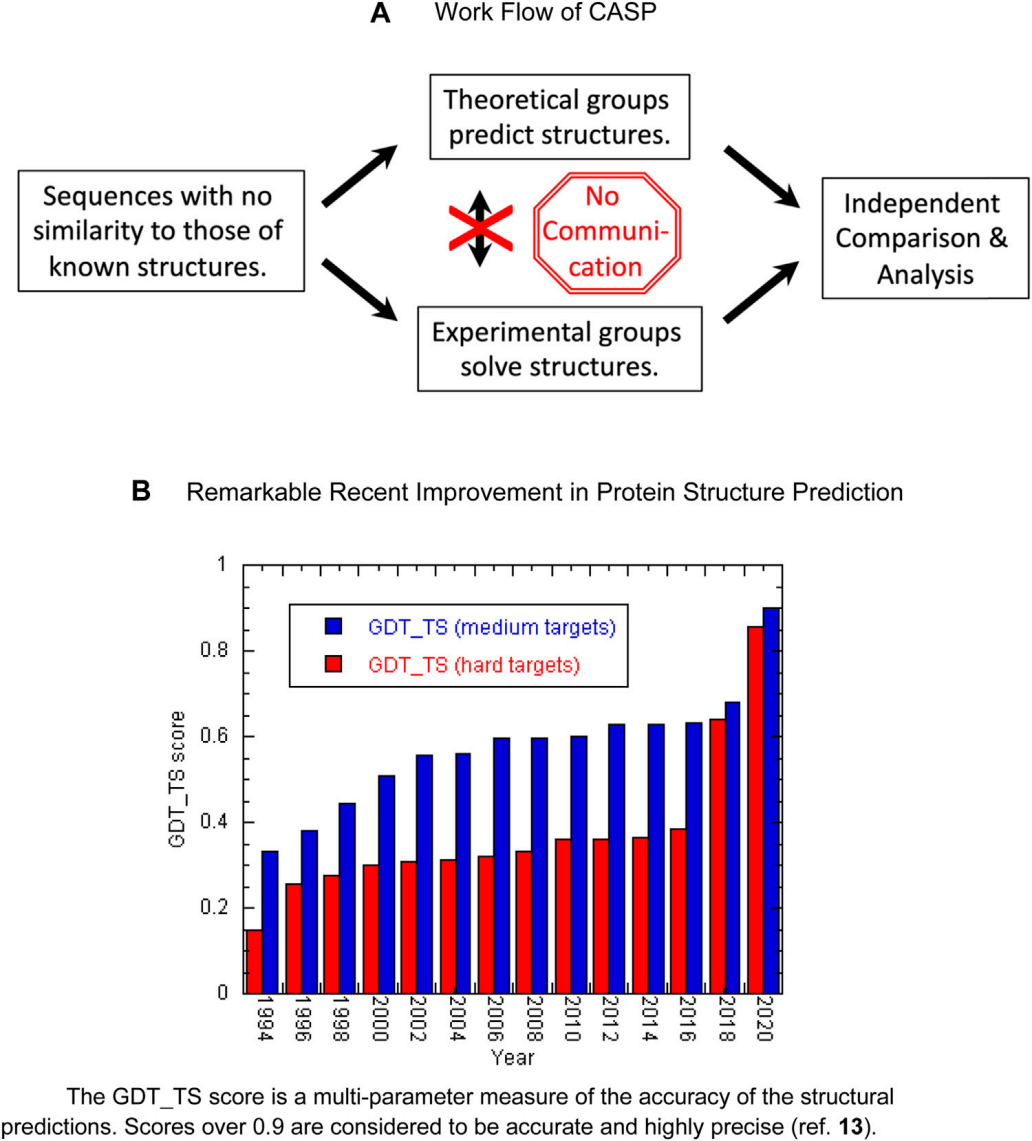
**(A)** Work flow of CASP. **(B)** Remarkable recent improvement in protein structure prediction. The GDT_TS score is a multi-parameter measure of the accuracy of the structural predictions. Scores over 0.9 are considered to be accurate and highly precise (ref. 13).

**FIGURE 2:**
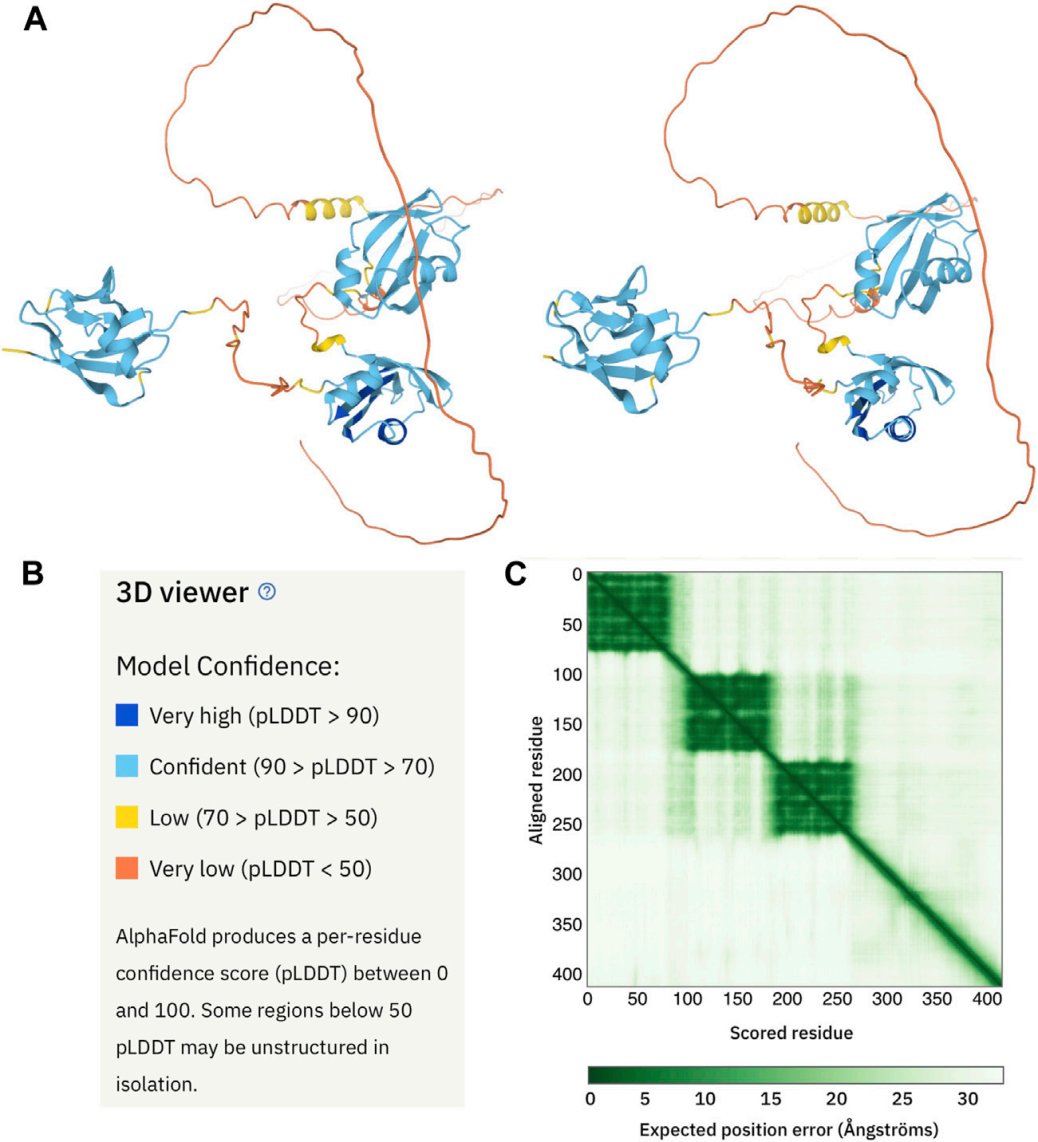
AlphaFold 2 structural model of human TDP-43. **(A)** Cross-eyed stereo view of the AlphaFold 2 output (https://alphafold.ebi.ac.uk/entry/Q13148) for a representative human protein, TDP-43. As defined by a color code panel **(B)** the protein contains well folded N-terminal (appearing on the left) and two RRM domain (center) followed by a long, poorly predicted region. The former are in good agreement with their NMR solution structures (Mompeán et al., 2016) (Lukavsky et al., 2013). The latter is known to be disordered but does contain a partly populated helix approximately in the position predicted with low confidence (yellow) by AlphaFold 2. The expected positional error panel **(C)** shows dark green patches for the three domains, indicating that they are well defined. Note that the green shading in weak between the N-terminal domain (residues 1-80) and the RRM domains (residues 103-260). This means that their inter-domain configuration is not well defined. By contrast, the green shading between the two RRM domains (residues 103- 175 and 195-260) is darker, indicated that their relative orientation is better defined.

Since 1994, a community experiment called “CASP” (critical assessment of methods for protein structure prediction) has provided a proving ground for algorithms trying to solve the protein folding problem (Moult et al., 1995). Researchers are given protein sequences unrelated to those of known structures. Then, they attempt to predict their structures while other groups experimentally determine the structures using X-ray crystallography or NMR spectroscopy (Figure 1A). Finally, the accuracy of the predictions are independently assessed. As described in detail in an excellent, recent account (Pearce and Zhang, 2021) one group of CASP participants researchers attempted to model the target proteins based on homology with known structures whereas a second group tried to construct protein structure models based on the physicochemical principles. From the mid-1990s until 2016, the accuracy of the predictions slowly improved, especially for structures considered to be moderately challenging, whereas difficult proteins remained intractable (Kryshtafovych et al., 2021) (Figure 1B). In 2018, and especially in 2020, however, significant improvements were seen thanks to the development and application of deep learning and artificial intelligence algorithms, particularly AlphaFold 2 (Kryshtafovych et al., 2021). AlphaFold 2 uses both protein sequences and structures as input to a multi-layered neural network (Jumper et al., 2021). Multiple sequence alignments reveal amino acids which co-evolve, inferring that they are in contact. For example, a contact could be inferred if two positions were found to have a statistical preference for residues that interact favorably, such as Glu and Lys. AlphaFold 2 also includes Amber refinement (Duan et al., 2003) as a last step. Remarkably, the program’s structural models accurately predict both backbone and side chain positions with a precision ≤  1Å (Jumper et al., 2021). In CASP14, some of the test structures were determined by NMR spectroscopy; one structure predicted with AlphaFold 2 actually agreed better with the NMR spectral data than the structure obtained from standard NMR data analysis and structure calculation (Huang et al., 2021). However, AlphaFold 2 did not succeed with intrinsically disordered proteins (Huang et al., 2021). A further strength of AlphaFold 2 is its speed; the prediction of a 400 residue protein structure requires only about one GPU minute (Jumper et al., 2021). This has enabled the method to be applied to whole proteomes of proteins and the structures predicted by AlphaFold 2 for the human proteome and 20 other proteomes are now publicly available (Tunyasuvunakool et al., 2021) at https://alphafold.ebi.ac.uk. High confidence predictions are reported for two-thirds of the proteome. While few percent of the regions or proteins predicted with low/very low confidence by AlphaFold 2 may be novel folds; the great majority corresponds to intrinsically disordered regions. The AlphaFold 2 output for a representative protein, TDP-43, with contrasting high confidence, folded domains and low confidence, disordered regions, is shown in Figure 2.

### AlphaFold 2 and Membrane Proteins

Membrane proteins have always challenged structural biologists. Although AlphaFold 2’s performance on water-soluble globular proteins is impressive, there are some doubts regarding its capacity to predict the structure of membrane proteins. Would the small fraction of membrane proteins in the PDB database (less than 3% of the total (Li et al., 2021) and https://blanco.biomol.uci.edu/upstruc), which was used to train AlphaFold 2, be sufficient for the program to capture concepts about their structures? Also, could AlphaFold 2 handle mobile protein regions which extend beyond the membrane proteins into the aqueous phase? Moreover, in a recent elucidation of a novel membrane protein called ChRmine, some structural features were reported to be mispredicted by AlphaFold 2 (Kishi et al., 2022). However, in a thorough retrospective study (Hegedűs et al., 2022), which used new membrane protein structures reported after the optimization and launch of AlphaFold 2, the program was found to be likely to perform as well with membrane proteins as with water soluble proteins. For both classes of proteins, disordered regions are modeled with low confidence and for the former they are sometimes incorrectly threaded through the membrane. Moreover, performance is worse when the membrane thickness is small, as was the case for ChRmine. Nevertheless, the overall performance of AlphaFold 2 on membrane proteins is excellent and it is particularly impressive considering that AlphaFold 2 training did not include an explicit lipid bilayer. Even homodimeric membrane proteins are reported to be correctly modeled, when inputted into the program as two copies of the monomer sequence connected by a linker sequence. Considering that membrane proteins represent less than 3% of PDB structures but compose over 27% of the proteome (Almén et al., 2009), the ability of AlphaFold 2 to accurately predict membrane protein conformations represents an important advance in Structural Biology.

### JMB Special Issue on AlphaFold 2… TLDR

Due to its profound impact on Biochemistry and Structural Biology, AlphaFold 2 has been the subject of commentary by authorities in the special volume edited by Serpell, Otzen and Radford (Serpell et al., 2021) of J. Mol. Biol., which appeared in October of 2021. Their main points are summarized in the following paragraphs.

A. Fersht compared and summarized Machine Learning (ML) programs for chess and Go to AlphaFold 2 (Fersht, 2021). Compared to older chess programs, such as Deep Blue which defeated Kasparov in the 1990s by a brute force approach of testing myriads of possible moves, newer ML programs are different. They master games by studying old games and playing them themselves. A key “sea change” moment for ML occurred in 2016 when the ML Go program “Alpha Go” invented a completely novel moves, some of which was seen at the moment as errors but proved to be strokes of genius as highlighted in the documentary movie AlphaGo (https://www.youtube.com/watch?v=WXuK6gekU1Y). Fersht notes AlphaFold 2 is an especially impressive achievement considering that for games like chess or Go, it was possible to program the rules, however for protein structure, AlphaFold 2 had to infer indirectly the rules from the protein sequences and structures. Fersht noted that while some professional chess players retired in the face of superior ML chess programs such as Alpha Go Zero, others use these programs as tools to better their game. Fersht proposes that we proteinologists follow their example and use AlphaFold 2 as a tool for protein design.

This thread is continued by D. Woolfson (Woolfson, 2021), who pointed out that α-helices are widely used in protein design because they are self-contained structural elements. He also emphasized the importance of “negative design”, that is, to disfavor unwanted conformations, in creating new protein structures. Considering that protein design has shown that the “space” of possible folds is much greater than what is observed in natural proteins, Woolfson points out that it will be interesting to see if AlphaFold 2 can “predict” the conformation of designed proteins whose structures are very different from those found in the protein database. The ability of AlphaGo to invent new moves in the game Go suggests AlphaFold 2 may have similar success in novel protein design. Other challenges for the future of protein design mentioned by Woolfson (Woolfson, 2021) include developing novel binding sites, catalysts and allostery.

M. K. Higgens addressed how and whether AlphaFold 2 can help advance structural biology questions related to pandemics such as SARS-CoV-2 (Higgins, 2021). Higgens points out that AlphaFold 2 is probably not the best tool for predicting how the mutations present in different strains affect the conformation of key viral proteins. For example, for the SARS-CoV-2 spike protein, during the development of the highly successful mRNA vaccines, it was key to develop an mRNA that coded a mutant spike protein that highly stabilized the “closed” form and not the post-fusion “open” form (Fauci, 2021). This is an issue that is thought to be challenging for AlphaFold 2, but there are other programs which are especially designed to predict the impact of mutations (for an example, see (Goldenzweig et al., 2016)). Higgens further remarked that AlphaFold 2 is probably not well suited to address the effect of glycosylation, which can modulate viral protein function and mask them from the immune system or to viral proteins fragmenting and then to adopting alternative conformations to perform distinct functions.

**FIGURE 3:**
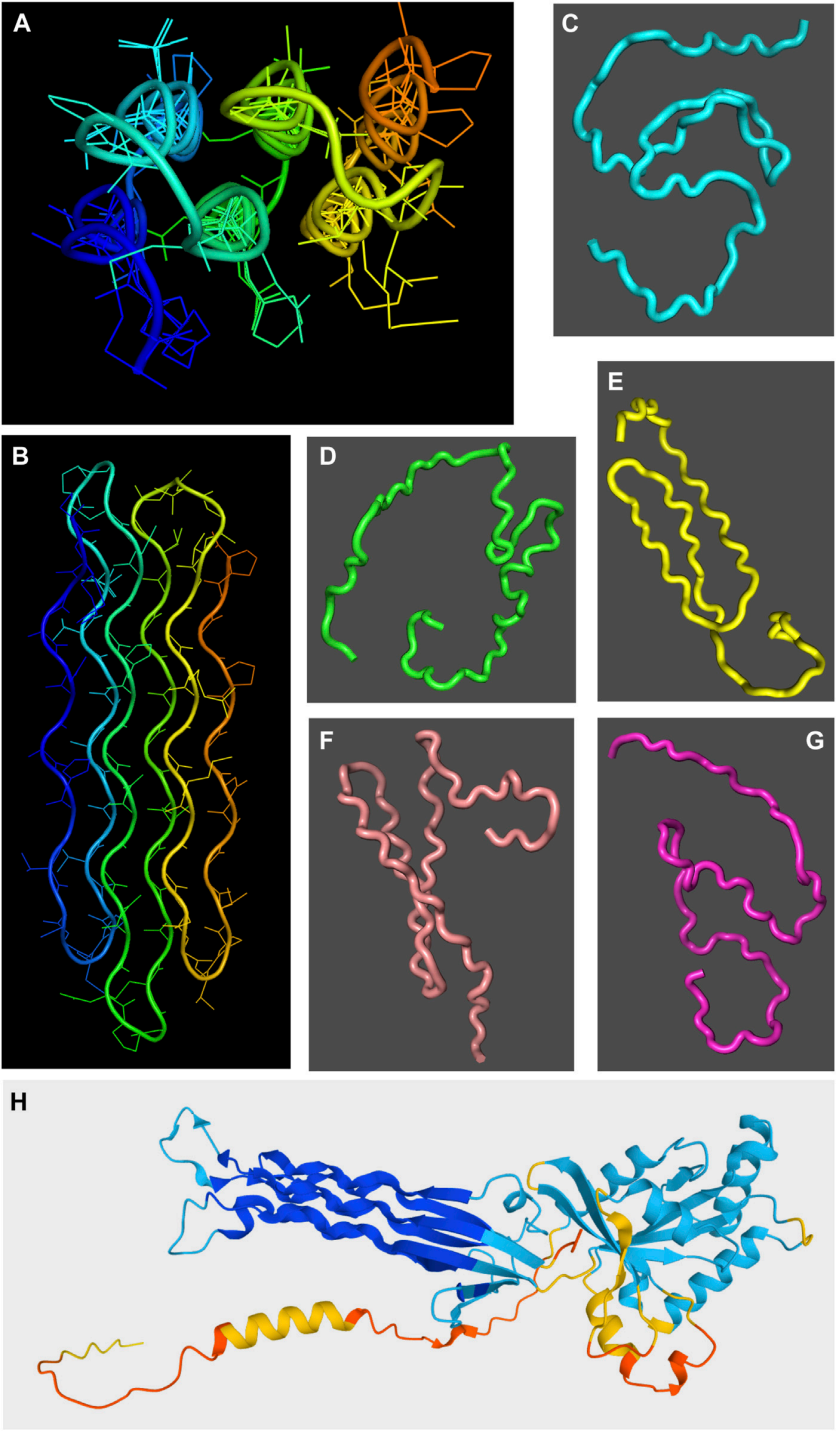
Chiaroscuro in AlphaFold 2 prediction of polyproline II helical bundle proteins. The snow flea antifreeze protein (sfAFP; PDB 3BOG) structure as solved by X-ray crystallography (Pentelute et al., 2008) is shown in two orientations in panels **(A,B)** with the chain colored in a rainbow blue to red spectrum from the N-terminus to the C-terminus. In panels **(C–G)**, five AlphaFold 2 structural models are shown for the sfAFP protein over a dark gray background. Whereas some polyproline II-like extended conformations are seen in structures shown in panels **(E,F)**, overall the method does not succeed in predicting sfAFP’s structure. By contrast, the essentially correct AlphaFold 2 structural model of the *E. coli* Obg GTPase is shown in panel **(H)**. The ribbon model is colored from blue (very high confidence) to red (very low confidence). The polyproline II helical domain, colored blue, is the region predicted with the highest confidence throughout the entire structure.

About a third of the human proteome is predicted with low or very low confidence by AlphaFold 2. K.M. Ruff and R. V. Pappu (Ruff and Pappu, 2021) pointed out that it is now widely believed that almost all these low/very low confidence regions are intrinsically disordered, with the remaining 1–2 % being well folded proteins which are mispredicted by AlphaFold 2. In our laboratory, we have found that AlphaFold 2 does not correctly predict the structure of a glycine-rich protein which adopts well folded polyproline II helical bundle (Mompeán et al., 2021) (Figure 3). Nonetheless, the regions marked by AlphaFold 2 as low/very low confidence are almost always disordered. This makes AlphaFold 2 one of the best algorithms for predicting disordered domains or proteins. Since AlphaFold 2’s approach is completely different from those of other disorder predictors, this strongly suggests that in the future improved hybrid methods can be developed. Profs. Ruff and Pappu also correctly point out that the extended spaghetto representation of low confidence regions is not an accurate representation of a disordered protein for a couple of reasons (Ruff and Pappu, 2021). First, it is well known that the disordered state ensemble is populated by a large number of transient conformations, not one extended structure. Secondly, the radius of gyration of the AlphaFold 2 representation is not accurate since many disordered regions show a wide variation of radii of gyration as shown by SAXS measurements (Ruff and Pappu, 2021). Disordered regions and proteins frequently adopt compact states especially when they undergo liquid/liquid phase separation. Thirdly, AlphaFold 2 always “bets on the favorite horse” so it does not serve to detect minor populations of structure or rigid conformations within a disordered protein, or zones that may fold under certain conditions or upon binding other proteins.

In their articles, B. Strodel (Strodel, 2021) and S. Ventura and coworkers (Pinheiro et al., 2021) described the challenges for AlphaFold 2 to address protein aggregation and amyloid structures. This is difficult for AlphaFold 2 for three reasons. Firstly, a large proportion of protein sequences can adopt an amyloid structure (Fändrich et al., 2001). This undermines the analysis of sequence data to obtain structural inferences. This is exacerbated by many amyloidogenic sequences being low complexity, consisting of stretches of a few different residues or just one residue, such as polyglutamine. Secondly, for the many amyloids which are pathogenic, not functional, their structures are decoupled from natural selection. This means that their sequences evolve in a more random way and can not provide structural inferences. Ventura and his team insightfully point out, however, that for functional amyloids, structural clues from sequence could be obtained. Thirdly, amyloids exhibit polymorphism, with the same sequence being capable of adopting diverse amyloid structures with different pathological outcomes, such as the distinct Tau amyloid structures seen in AD, FTD and “boxer’s dementia” (Shi et al., 2021). The study of the bases of these distinct amyloid structures is still an active and growing field (Shi et al., 2021) (Lövestam et al., 2022). B. Strodel also advanced that many of these gaps in AlphaFold 2’s capacities can be filled by molecular dynamics (Strodel, 2021). Finally, Ventura and coworkers noted that most predictive algorithms of amyloid forming stretches look for partially exposed hydrophobic segments, but buried hydrophobic segments in folded proteins can also form amyloid if they become exposed. Predicting such hidden amyloidogenic segments accurately relies on having a precise protein structure. By providing more precise protein structural models, Ventura and coworkers predict that AlphaFold 2 could be harnessed to improve the prediction of amyloid formation (Pinheiro et al., 2021).

Other computational approaches can also be applied to extend AlphaFold 2 structural models. For example, Normal Mode Analysis, which treats a protein structure as an oscillating system moving sinusoidally about a ground state, is computationally inexpensive and can reveal the effects of ligand binding and allosteric states (Wako and Endo, 2017). By contrast, Monte Carlo approaches probe protein structure through random sampling and statistical analysis. They can provide information on thermodynamics and folding kinetics of individual residues (Heilmann et al., 2020). Other Monte Carlo experiments have been used to characterize the conformational ensemble of intrinsically disordered proteins (Ciemny et al., 2019).

## New Horizons

Protein electrostatics is another important area where AlphaFold 2 has been combined with another method to achieve advances. Alone, AlphaFold 2 does not predict the pKa or charged state of the titratable residues like Asp, His or Glu. This information is very important for assessing electrostatic interactions, solubility and binding to macromolecules, substrates and drugs, but experimental pKa measurements are generally laborious (Laurents et al., 2003). Fortunately, a rather successful empirical method for estimating pKas is available called PropKa (Li et al., 2005); it uses a protein 3D structure and takes into account factors like burial, which tends to favor the neutral state and the proximity of other charged groups to calculate approximate pKa values. Thanks to AlphaFold 2, the availability of accurate 3D structures has now enabled the complete calculation of all titratable residues in the whole human proteome (Chen et al., 2022).

### AlphaFold 2 and Protein Complexes

Although AlphaFold 2 was developed to predict monomeric protein structures, in many cases it can be tricked into calculating the structure of dimers. This is done by putting both protein sequences into the same input file separated by a dummy sequence that acts as a flexible linker (Bryant et al., 2022); the latest on-line versions of AlphaFold 2, such as the colab notebook AlphaFold.ipynb, allow multimer structures to be predicted by separating the PDB filenames by colons. This approach works well when two or a few proteins interact like a faithful matrimony so that the residues lining the binding surface undergo co-evolution. However, when a protein has many binding partners, inference from multiple sequence alignments and co-evolution becomes blurred, impacting the prediction. Currently, research is underway to combine AlphaFold 2 with experimental methods like cryoelectron microscopy (CryoEM, *vide infra*), or other computational tools such as RoseTTAFold developed by D. Baker and his laboratory (Humphreys et al., 2021) to predict the structure of protein complexes. In particular, the combination of AlphaFold 2 with alternative multiple sequence alignments has been recently reported by A. Elofsson and his team to be the best approach for predicting heterodimeric protein complexes and discriminating non-binders (Bryant et al., 2022).

### AlphaFold 2 Complements Experimental Approaches For Structure Determination

AlphaFold 2 can enhance low and medium resolution biophysical methods. By providing highly accurate structural models, AlphaFold 2 could aid the interpretation of circular dichroism or fluorescence spectra. Imagine, for example, a protein with three Trp residues which shows fluorescence spectral changes upon ligand binding. By revealing that two Trp residues are completely buried and that the third is on the surface, AlphaFold 2 could putatively identify which Trp is at the binding site.

For crystallography, structural models afforded by AlphaFold 2 can be utilized to calculate phases by molecular replacement to solve protein structures using experimental X-ray diffraction data (Millán et al., 2021), and represents a moderate improvement relative to current protocols based on homology (McCoy et al., 2022). In particular, for 34 test cases, AlphaFold 2 derived molecular replacement phases led to the successful elucidation of 31 structures; the cases where it did not work involved proteins with long α-helices with small kinks that gave rise to large displacements (McCoy et al., 2022).

Cryo-EM excels at determining the structure of enormous protein complexes. Although improving, its resolution is often in the 3–4 angstrom range and can vary throughout a large structure. In these cases, as exemplified by the very recent elucidation of the large multidomain non-structural protein 2 (NSPS 2) from SARS-CoV-2 (Gupta et al., 2021), or modeling of the truly gigantic nuclear pore complex (Mosalaganti et al., 2021), AlphaFold 2 can serve as a useful complement by providing high resolution structural models which can be fit into the cryoEM electron density map. Taking this line of research one step further, F.J.B. Bäuerlein and W. Baumeister propose combining AlphaFold 2 structural models with cryoEM tomography results for “visual proteomics” (Bäuerlein and Baumeister, 2021), which integrates high resolution and medium resolution data from different approaches to provide a holistic view of organelles and cellular machinery. Besides cryoEM, other methods like cryo-electron tomography, which can provide medium (up to 4–5 Å) resolution protein structures in an unpurified cellular context (Ni et al., 2022)**,** and cryoEM microcrystal electron diffraction, which yields high resolution structures of proteins from crystals far too small for standard X-ray diffraction methods (Nannenga and Gonen, 2019), are maturing rapidly.

Whereas X-ray crystallography and CryoEM obtain data on proteins in highly non-physiological conditions; namely extremely low temperatures and/or trapped inside crystals, NMR spectroscopy can provide high resolution structural data under near physiological conditions of pH, concentration and temperature. Therefore, as pointed out by M. Zweckstetter, it is important to use NMR spectroscopy to assess how accurately AlphaFold 2 structural models represent the structure of proteins in solution (Zweckstetter, 2021). For three small, stable, well-folded proteins, excellent agreement was found for the NMR solution structures and the AlphaFold 2 structural models (Zweckstetter, 2021). By contrast, another recent study suggested that for many proteins, AlphaFold 2 structures are generally more precise and accurate than those solved by NMR spectroscopy (Fowler and Williamson, 2022). In particular, proteins with long loops that tend to yield few constraints, such NOEs, for NMR structural calculations are generally excessively floppy and are better determined by AlphaFold 2. However, for about 3% of the 904 cases studied, NMR spectroscopy produced superior results by detecting small elements of secondary structure or kinks in α-helices that are missed by AlphaFold 2 (Fowler and Williamson, 2022). NMR spectroscopy is also better at characterizing rare or alternative conformations or disordered regions as will be discussed in more detail below.

In assessing the relative accuracy of AlphaFold 2 and NMR structures, it is important to consider that AlphaFold 2 draws much insight from the PDB, which is dominated by X-ray crystal structures (92% of the total) compared to NMR (8%). The very cold temperatures typically used by X-ray crystallography rigidify protein structures (Tilton et al., 1992), especially the loops (Tilton et al., 1992). Moreover, crystal packing tends to stiffen loops and limit their conformational diversity (Eyal et al., 2005). This might bias AlphaFold 2 structural models to have overly rigid loops. To address this issue, Williamson and coworkers recently proposed comparing the flexibility of a protein calculated from its backbone ^1^HN, ^15^N, ^13^Cα, ^1^Hα, ^13^C, and ^13^Cβ nuclei to the flexibility calculated from the protein structure using mathematical rigidity theory (Fowler et al., 2020). They found that whereas X-ray crystal structures tend to model loops too rigidly, the loops in NMR structures are too floppy. These findings should be useful to fine tune the definition of loops in future versions of AlphaFold and other ML protein prediction programs.

**FIGURE 4:**
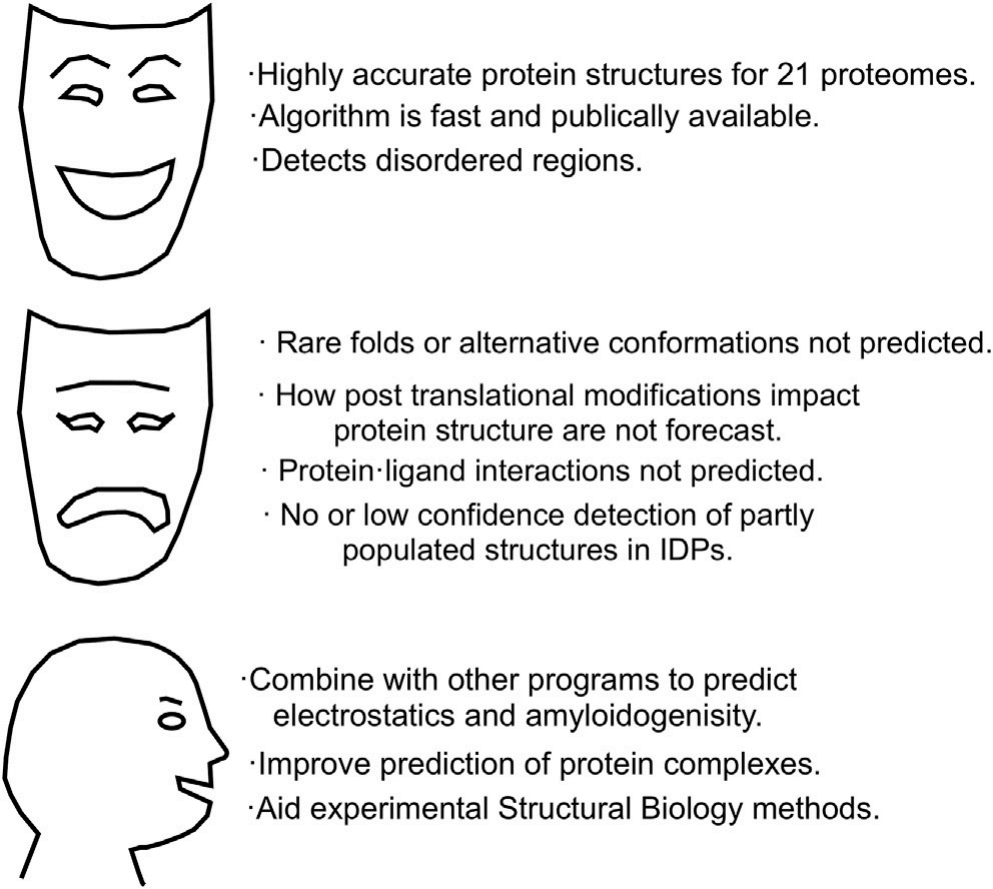
AlphaFold 2. Strengths, weaknesses and future directions.

Taken together, these commentaries suggest there is some broad agreement on the AlphaFold 2’s strengths, its weaknesses and the new horizons it opens up, which are summarized in Figure 4.

### NMR Spectroscopy Can Help With AlphaFold 2’s Shortcomings

In the previous paragraphs, ways that AlphaFold 2 could advance NMR spectroscopy and other structural biological methods were described. Regarding the weaknesses of AlphaFold 2, some of them, such as the prediction of protein complexes and the prediction of highly unusual folds, may well be overcome in the next few years. However, four weaknesses seem to be more difficult for AlphaFold 2, or ML in general, to overcome. These are: 1) the prediction of folds that populate only a small fraction of a conformational ensemble, 2) the impact of post translational modifications, 3) the prediction of interaction with ligands and 4) the prediction of partially populated structure in intrinsically disordered proteins. Fortunately, these problems can be addressed using NMR spectroscopy.

**FIGURE 5:**
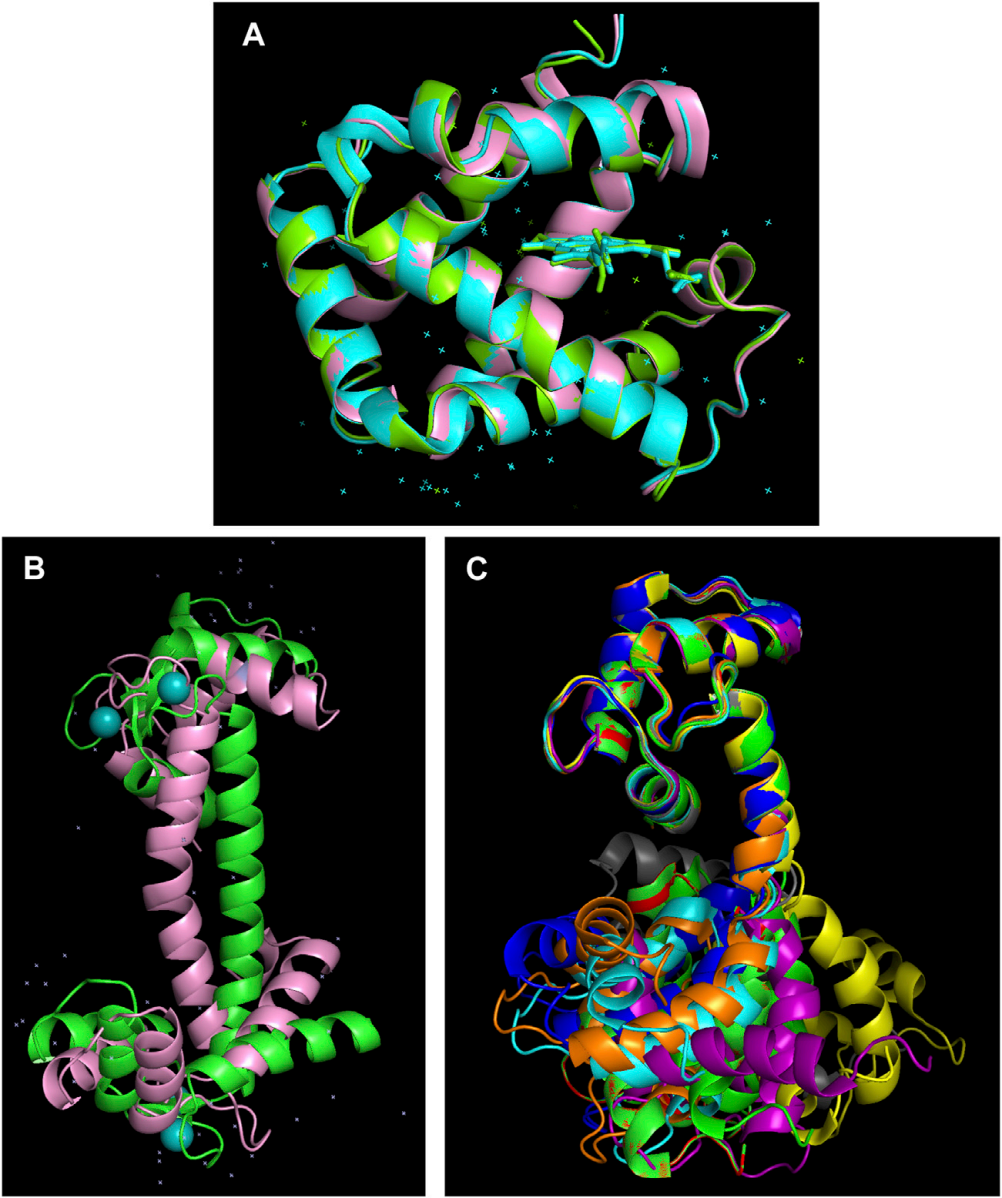
AlphaFold 2 overlooks alternative conformations. **(A)** The human hemoglobin α-subunit in the tense (cyan, PDB 1VWT) and relaxed (green, PDB 1RVW) forms solved by X-ray crystallography (Puius et al., 1998) superimposed on the AlphaFold 2 predicted structure (pink, https://alphafold.ebi.ac.uk/entry/P69905 ). Despite no heme group or water molecules being present in the AlphaFold 2 input, the correct structure is computed with an RMSD of < 0.5 angstroms. **(B)** Calmodulin contains two well folded domains connected by a long, unstable α-helix. Bending in the central α-helix of the AlphaFold 2 model (https://alphafold.ebi.ac.uk/entry/P0DP23) leads to large RMSD values relative to the X-ray crystallography structure of the Ca^++^-bound form of calmodulin (PDB 3CLN) (Babu et al., 1988). **(C)** NMR solution structures reveal that the central α-helix is disordered in the absence of Ca^++^ provoking significant conformational diversity which is highly relevant for the protein’s biological function (Zhang et al., 1995) (Kainosho et al., 2006).

As mentioned previously, AlphaFold 2 always bets on the winning horse, meaning that it predicts the most likely structure. However, protein molecules are constantly sampling alternative conformations and even unfolded states as their conformational stability is marginal (Pace and Hermans, 1975). These alternative conformations can be subtly different, such as the tense and relaxed conformations of hemoglobin with distinct oxygen affinities (Figure 5A) or more notorious, like the apo- and Ca^++^-bound forms of calmodulin (Figures 5B,C). The distinct conformations of these proteins is physiologically vital, but AlphaFold 2 only predicts one of them (Figure 5). X-ray crystallography (Puius et al., 1998) and NMR spectroscopy (Zhang et al., 1995) (Kainosho et al., 2006) are able to characterize alternative conformations.

Rare conformations can also be important in health and disease. NMR-monitored hydrogen/deuterium (H/D) exchange provided the first way to detect and characterize sparsely populated intermediates forming during protein folding (Udgaonkar and Baldwin, 1988). H/D exchange measurements can also be used to probe slow conformational changes, like the unfolding and refolding of α-helices and β-strands (Mayo and Baldwin, 1993) (Skinner et al., 2012) as well as to measure protein conformational stability (Huyghues-Despointes et al., 1999). Protein H/D exchange can also be measured by mass spectrometry (Wales and Engen, 2006). Compared to NMR, mass spectrometry is not a real time experiment, but it can be automated and a ^15^N-labeled sample and an assigned ^1^H-^15^N HSQC spectrum are not prerequisites.

In the last 15 years, a new series of NMR experiments have been developed, i.e., dark state exchange saturation transfer, Carr-Purcell-Meiboom-Gil relaxation, chemical exchange saturation transfer, paramagnetic relaxation enhancement, which reveal conformational populated to a few percent forming under different time scales (Sekhar and Kay, 2013) as well as residual dipolar couplings which show the relative orientation of segments (Zweckstetter, 2021). These methods are being used to characterize conformations with a small population making a big impact in enzymatic action (Sekhar and Kay, 2013), ion-channel regulation (Bernardo-Seisdedos et al., 2018) and amyloid formation (Fawzi et al., 2011). If the results from these NMR experiments are deposited in well-curated databases, it might eventually be possible to apply ML and use them to obtain correlations with AlphaFold 2 predicted structures with sub-optimal scores. For example, AlphaFold 2 models could be used to initiate MD simulations or related computational methods to sample rare conformations of biological interest. In this way ML methods could be leveraged to predict and identify rare structures.

Post translational modifications (PTM) are quite common in proteins; over 10% proteins are phosphorylated and another 10% plus are glycosylated (Khoury et al., 2011). PTMs are also quite diverse; over 400 different types of modifications are known (Ramazi and Zahiri, 2021). Protein structure can be strongly impacted by these modifications. For example, the phosphorylation of one serine residue in the RNA-binding K-homology splicing regulator protein (known to impact interactions with partners and mRNA degradation) was found by NMR spectroscopy to act by provoking the unfolding a key domain (Díaz-Moreno et al., 2009). Glycosylation is probably the most complex class of PTMs due to the great number of different sugars and branching patterns. In SARS-CoV-2, both the spike protein and its receptor protein are glycosylated and NMR spectroscopy has revealed the identity and diversity of their oligosaccharide chains (Lenza et al., 2020). For phosphorylation and glycosylation as well as other PTMs, AlphaFold 2 unfortunately does not consider their impacts on protein structure. However, there are a score of databases on protein PTMs and computational tools for predicting PTMs (Ramazi and Zahiri, 2021). Therefore, once sufficient knowledge on how PTMs affect proteins is acquired from NMR spectroscopy and other experimental methods such as mass spectrometry, it may eventually become possible to create new algorithms to forecast such impacts. These new algorithms might then be applied to AlphaFold 2 generated structures and PTM databases to achieve a holistic approach to protein structure and PTM prediction.

The pharmaceutical industry uses protein structures as a starting point for screening compound libraries to identify ligands as lead compounds for drug development. The enormous increase in accurate protein structures provided by AlphaFold 2 will certainly stimulate these efforts, however, enzyme active sites tend to “break the protein folding rules” and have unusual conformations (Mullard, 2021). This casts doubts on the utility of AlphaFold 2 for drug discovery (Mullard, 2021). One application of NMR spectroscopy is to determine protein structures. It is especially useful for small proteins while studies of larger proteins becomes tedious and expensive as larger magnets, deuteration and selective methylation are necessary to overcome signal overlap and resonance broadening from slow tumbling (Frueh et al., 2013). A second application of NMR, which is gaining in importance, is to detect and characterize the binding of drug-like ligands.

In a first round of screening experiments, 1D ^1^H NMR experiments can be performed on a mixture of several binding candidate molecules in the presence of dilute, unlabeled protein. These experiments detect changes in the chemical shift and resonance width of the ligands or in their dynamics, as a compound bound to a protein will show faster transverse (T_2_) relaxation, or in their diffusion rate, since a small molecule united to a larger protein will diffuse more slowly (Hajduk et al., 1997). Another powerful approach is saturation transfer difference (STD) NMR spectroscopy, wherein a large molecule is excited selectively and then some of the magnetization is transferred to the bound ligand. In many cases, when binding is not too tight or too weak, the dissociation constant and the part of the ligand that binds can be determined (Walpole et al., 2019). The WaterLOGSY NMR approach transfers magnetization from water to protein and then to ligand, thus revealing changes in solvent accessibility of the free versus protein bound ligand (Raingeval et al., 2019). By performing all these fast 1D ^1^H based NMR experiments, up to five different lines of evidence for binding can be quickly obtained. Once a ligand is identified by these fast methods, binding can be further corroborated and the binding site in the protein can be mapped using 2D ^1^H-^15^N HSQC NMR spectroscopy. This experiment requires an assigned ^1^H-^15^N HSQC spectrum and also serves to study protein/protein or protein/nucleic acid interactions (Zuiderweg, 2002).

In addition to the nuclei (^1^H, ^13^C and ^15^N ) which are typically observed in NMR studies of proteins, two other spin ½ nuclei, ^19^F and ^31^P are useful for binding studies. Due to its very wide range of chemical shift values and the sensitivity of its resonance to broadening upon interaction, ^19^F has been incorporated into chemical libraries to facilitate the screening of small drug-like “fragment” compounds (Troelsen et al., 2020). On the other hand, a protocol based on a modified genetic code has enabled the site-specific labeling of a large membrane protein with ^19^F (Wang et al., 2021). Its signal in the presence or absence of ligand proved crucial to identify an allosteric binding site (Wang et al., 2021). ^31^P NMR can be exploited to study protein phosphorylation (Hirai et al., 2000) as well as characterize the conformation and dynamics of nucleic acids (Saxena et al., 2015).

About a third of eukaryotic proteins are intrinsically disordered or contain a disordered region at least thirty residues long. Since their discovery (Pontius and Berg, 1990) intrinsically disordered proteins (IDPs) have been found to play numerous physiological functions including the integration of numerous and diverse clues in protein signaling networks (Uversky, 2016). As mentioned previously, the low sequence complexity and conservation and a lack of stable structures complicates their analysis by ML. The conformation and biological activities of IDPs are also highly affected by PTMs which adds further difficulty to their study. NMR spectroscopy has been the key experimental method for obtaining atomic level information on IDPs (Dyson and Wright, 2021). Disordered regions can also act as association tags that enable proteins to form biomolecular condensates. This occurs for the disordered region of TDP-43 shown in Figure 2 (Conicella et al., 2016). IDPs frequently contain partly populated conformations or segments that become well folded when they bind to a partner. For example, certain segments of the IDP Tau tend to form α-helices and β-strands (Mukrasch et al., 2009). The population of two β-strands increases when Tau binds to μtubules (Kadavath et al., 2015). However, they can also become fully structured in the Tau amyloid structures associated with Alzheimer’s disease and other dementias (Lövestam et al., 2022). AlphaFold 2 does not serve for characterizing IDPs. Nevertheless, the DisProt database is consolidating knowledge on IDPs (Quaglia et al., 2022) and it is possible that some future ML program may learn to glean conformational inferences from experimental data, thus paving the way to IDP characterization by ML and other computational approaches (Lindorff-Larsen and Kragelund, 2021).

In conclusion, AlphaFold 2 accurately and precisely predicts the structure of well-folded proteins. Moreover, it can be combined with other computational methods to advance our understanding of protein electrostatics and forecast protein complexes. However, it does not predict rare conformations, the impact of post translational modifications (PTM), ligand binding or partially structured zones in intrinsically disordered proteins (IDPs). Fortunately, these shortcomings of AlphaFold 2 are strengths of NMR spectroscopy. In the future, results from NMR spectroscopy and other experimental methods could pave the way for future ML methods able to predict sparsely populated conformations, the effects of PTM, small molecule binding and preferred conformations in IDPs.
